# HTLV-1 Tax: centrosome amplification and cancer

**DOI:** 10.1186/1742-4690-3-50

**Published:** 2006-08-09

**Authors:** Anne Pumfery, Cynthia de la Fuente, Fatah Kashanchi

**Affiliations:** 1 Seton Hall University, Department of Biology, South Orange, NJ 07079, USA; 2 The Rockefeller University, Laboratory of Virology and Infectious Disease, New York, NY 10021, USA; 3 The George Washington University Medical Center, Department of Biochemistry and Molecular Biology, Washington, DC 20037, USA; 4 The Institute for Genomic Research, Rockville, Maryland 20850, USA

## Abstract

During interphase, each cell contains a single centrosome that acts as a microtubule organizing center for cellular functions in interphase and in mitosis. Centrosome amplification during the S phase of the cell cycle is a tightly regulated process to ensure that each daughter cell receives the proper complement of the genome. The controls that ensure that centrosomes are duplicated exactly once in the cell cycle are not well understood. In solid tumors and hematological malignancies, centrosome abnormalities resulting in aneuploidy is observed in the majority of cancers. These phenotypes are also observed in cancers induced by viruses, including adult T cell lymphoma which is caused by the human T cell lymphotrophic virus Type 1 (HTLV-1). Several reports have indicated that the HTLV-1 transactivator, Tax, is directly responsible for the centrosomal abnormalities observed in ATL cells. A recent paper in *Nature Cell Biology *by Ching *et al*. has shed some new light into how Tax may be inducing centrosome abnormalities. The authors demonstrated that 30% of ATL cells contained more than two centrosomes and expression of Tax alone induced supernumerary centrosomes. A cellular coiled-coil protein, Tax1BP2, was shown to interact with Tax and disruption of this interaction led to failure of Tax to induce centrosome amplification. Additionally, down-regulation of Tax1BP2 led to centrosome amplification. These results suggest that Tax1BP2 may be an important block to centrosome re-duplication that is observed in normal cells. Presently, a specific cellular protein that prevents centrosome re-duplication has not been identified. This paper has provided further insight into how Tax induces centrosome abnormalities that lead to ATL. Lastly, additional work on Tax1BP2 will also provide insight into how the cell suppresses centrosome re-duplication during the cell cycle and the role that Tax1BP2 plays in this important cellular pathway.

## Background

Faithful duplication of the genetic content of cells and proper segregation into two daughter cells are two highly regulated and distinct processes. The cell cycle determines when cells will duplicate their genome and the four phases are regulated by a series of different cyclin/cyclin-dependent kinase complexes. Duplication of the genome occurs during the S phase while segregation of duplicated chromosomes into daughter cells occurs during the M (mitotic) phase. Proper segregation of chromosomes during M phase requires that the centrosomes are faithfully duplicated prior to mitosis. Duplication of centrosomes occurs at the end of G1 and the beginning of the S phase, yet is regulated separately from the duplication of the genome. Dysregulation of either the cell cycle or centrosome duplication can result in major genetic alterations and cancer [1-3]. Changes in centrosome duplication have been observed in cancers caused by genetic mutations as well as in cancers induced by viruses. A recent report by Ching *et al*. [4] further elucidates how the HTLV-1 Tax protein may modulate centrosome duplication resulting in aneuploidy and the development of adult T cell leukemia (ATL).

The centrosome functions as the microtubule organizing center (MTOC) of the cell and modulates the microtubule network critical for chromosome segregation, cell division, intracellular transport, and development [1-3]. The centrosome is composed of a pair of centrioles surrounded by the pericentriolar material [1,2], which is composed of at least 50 different proteins including several different tubulins, centrin, and proteins containing a coiled-coil motif [2]. To prepare for chromosome segregation in the next M phase, a single centrosome needs to be duplicated. During the G0 and G1 phases of the cell cycle there is a single centrosome that is inherited from the mother cell during the previous cell division, which is duplicated during the late G1 and S phases. During mitosis, the number of spindle poles is generally determined by the number of centrosomes [1].

Centrosome duplication occurs in a semi-conservative manner and consists of four phases: (i) centriole splitting in which the mother and daughter centrioles detach; (ii) semiconservative centrosome duplication, where a new centriole is formed adjacent to the original centriole; (iii) maturation involves recruitment of pericentriolar material proteins; and (iv) centrosome separation during mitosis to form the spindle poles [1,2]. Centrosome duplication is regulated by a series of phosphorylation and dephosphorylation events mediated by several kinases including Cdk2, Nek2, polo-like kinases, and aurora kinases [1,2,5]. The coordination between replication and centrosome duplication suggests that these events may be regulated by a common mechanism. Cdk2 is attractive as the central modulator since it is involved in both processes. Cdk2 is activated by binding to Cyclin E or Cyclin A at the start of S phase to induce DNA replication. Additionally, Cdk2 is required for centrosome duplication in a variety of cell systems [2,6]. Centrosomes are duplicated only once during each cell cycle and there is an apparent intrinsic block to reduplication of centrosomes [7]. However, the nature of this block is not known, but it apparently is overcome in cancer. Increased centrosome numbers leads to disturbances in mitosis and cytokinesis leading to errors in chromosome segregation and chromosome instability [6].

Alterations in centrosome duplication and the resulting aneuploidy are observed in a significant number of cancers. There are several possible mechanisms of centrosome amplification, including: (i) centrosome duplication more than once in a cell cycle; (ii) failure to undergo cytokinesis resulting in doubling of the genome [6]; and improper splitting, or fragmentation, of centrosomes in the M phase due to DNA damage [8]. Centrosome amplification is frequently seen in a number of solid cancers including breast, lung, colon, prostate, testicular, and head and neck squamous cell carcinoma [6], as well as several hematological malignancies such as Hodgkin's lymphoma, multiple myeloma, and chronic lymphocytic leukemia [9]. Some of the common genetic alterations in cancer associated with centrosome amplification include inactivation of p53, over-expression of Cyclin E [6], and mutation of BRCA1 [6,10].

Centrosome abnormalities are also observed in cancers caused by tumor viruses such as hepatitis B virus (HBV) [11-13], human papillomavirus [14,15], Kaposi's sarcoma-associated herpesvirus (KSHV) [16,17], and human T cell lymphotropic virus type 1 (HTLV-1) [4,18-20] [Table 1]. The HBV X protein induces centrosome amplification by activating the Ras-MEK-MAP kinase pathway [13]. Additionally, HBV X targets the Crm1 nuclear receptor, sequestering it in the cytoplasm [12]. Inactivation of Crm1 leads to abnormal centrioles and the presence of more than two centrosomes in 39% of mitotic cells [11]. For human papillomavirus, both the E7 and E6 proteins contribute to supernumerary centrosomes. E7 inactivates Rb [14] and induces an increase in the number of centrioles, a portion of which give rise to mature centrosomes [15], whereas E6 inactivates p53 [14]. Expression of either protein alone will induce centrosome amplification; however, co-expression of E6 and E7 results in a higher incidence of supernumerary centrosomes [3,14]. Similar to HPV E6, the KSHV latency associated nuclear antigen (LANA) binds to p53 and inhibits the ability of p53 to transactivate cellular genes resulting in abnormal centrosomes, multinuclear cells, and other genomic abnormalities [17].

**Table 1:** Effect of various viral proteins on centrosomal abnormalities

Viral Protein	Cellular Interacting Protein	Function in Normal Cells	Phenotype
HBV X	Crm1	Nuclear export receptor	Abnormal centrioles >2 centrosomes per cell
HPV E7	Rb	Tumor suppressor Transcriptional repressor	>2 centrosomes per cell
HPV E6	P53	Tumor suppressor	>2 centrosomes per cell
KSHV LANA	P53	Tumor suppressor	Abnormal mitotic spindles >2 centrosomes per cell
HTLV Tax	TaxBP181	Mitotic spindle checkpoint protein	Multinucleated cells
	RanBP1	Regulator of Ran-GTPase pathway	>2 centrosomes per cell
	Tax1BP2	Regulates centrosome duplication	>2 centrosomes per cell

## Discussion

The HTLV-1 Tax protein contributes to the ability of the virus to cause ATL by inactivating p53 [21-24], altering the cell cycle [25,26], and inactivating Rb [27]. As Tax is able to deregulate cellular checkpoints, most notably the G1/S transition [28,29], it is not surprising that this oncoprotein is involved in mitotic disruption. An early report demonstrated that expression of Tax alone was sufficient to induce both centric (containing a kinetochore and representative of segregation defects) and acentric (representative of DNA damage) micronuclei [30]. While clastogenic effects induced by Tax appears to be due to subversion of DNA damage repair pathways [31-34], Tax involvement in aneugenic damage was less clear. A study by Jin *et al*. [20] indicated that the association of Tax with hsMAD1, a mitotic spindle checkpoint (MSC) protein, led to the translocation of both MAD1 and MAD2 to the cytoplasm. The MSC is necessary to halt the cell cycle when an error(s) in chromosome segregation is detected. When all of the kinetochores are attached to both poles of the mitotic spindle apparatus and chromosomes are equally segregated to opposite poles, the block is then released [35]. By disrupting normal localization of these factors (*i.e*. MAD1 and MAD2) to kinetochores following chromosomal missegregation, Tax was able to usurp this checkpoint and thus allow for accumulation of multinucleated cells, a common phenotype of ATL cells. However, several questions remained including (i) was aneuploidogenic damage simply the accumulation of natural occurring mis-segregation events [36] due to Tax or (ii) were there other Tax-related mechanisms either direct or indirect, that induced aneugenic damage?

Evidence for the direct involvement of Tax in aneuploidy development was recently shown by Peloponese and colleagues [19]. Tax was shown to localize to centrosomes during mitosis and interact with the Ran-GTPase pathway through the Ran-binding protein 1 (RanBP1). This protein complex regulates mitotic centrosome stability and microtubule nucleation and spindle formation [37,38]. Interaction of Tax with RanBP1 was shown to be necessary for Tax targeting to centrosomes and induction of supernumerary centrosomes. The interaction of Tax with the Ran-GTPase pathway to dysregulate centrosome duplication is reminiscent of HBV X interaction with Crm1 [12], a Ran-GTP binding nuclear export receptor [39].

The involvement of Tax in centrosome amplification has been further strengthened by recent findings from Ching *et al*. [4]. Tax was observed to localize to centrosomes in both transfected cells and HTLV-1 transformed cells. About 30% of ATL cells displayed centrosome amplification (>2 centrosomes) and 30–80% of cells expressing Tax alone had supernumerary centrosomes indicating that Tax was responsible for the abnormal centrosome phenotype in ATL. Through a yeast two-hybrid approach, Tax was shown to associate with a coiled-coil protein, Tax1BP2 (formerly known as TaxBP121). Tax1BP2 has high sequence similarity to C-Nap1 (centrosomal Nek2-associated protein 1), a protein involved in centrosome cohesion during the interphase of cell cycle [40], and was shown to be part of the centrosome complex. Tax interaction with Tax1BP2 was shown to be necessary for Tax to induce supernumerary centrosomes and over-expression of Tax1BP2 countered this effect. Additionally, down-regulation of Tax1BP2 expression by siRNAs resulted in amplification of centrosomes leading the authors to speculate that Tax1BP2 may be the intrinsic block to re-duplication proposed by Wong and Stearn [7]. This interaction provides a novel way for Tax to induce aneuploidogenic damage through centrosome amplification. Furthermore, if Tax1BP2 proves to be important for the block to centrosome duplication this will be the first protein identified to perform such a function. However, several questions remain: (i) is there differential expression or localization of Tax1BP2 during the cell cycle; (ii) does knock-down of Tax1BP2 expression in the S and G2 phases allow for centrosome re-duplication; and more importantly (iii) how does Tax1BP2 function to limit amplification of centrosomes?

## Conclusion

**Figure 1:**
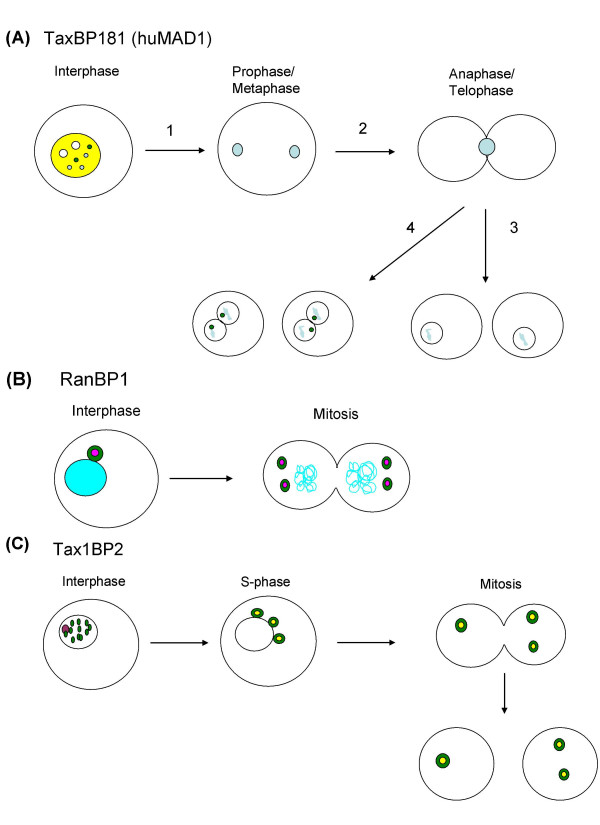
**Role of Tax binding proteins in centrosomal abnormalities observed in ATL cells**. **(A) TaxBP181 (hsMad1)**. During interphase TaxBP181 (light blue) is localized to the nucleus (yellow) and some co-localize with Tax (green) in infected cells. During the transition to prophase (1) in normal cells, TaxBP181 localizes to the kinetochores of chromosomes and allows proper segregation. (2) In normal cells during anaphase and telophase, TaxBP181 localizes to the midbody and finally in newly formed progeny nuclei. (3) When Tax is expressed in ATL cells, TaxBP181 is translocated to the nucleus, allowing for chromosome missegregation, resulting in multinucleated cells (4). **(B) RanBP1**. During interphase, Tax (green) is found in the nucleus and a portion is co-localized with RanBP1 (purple) on centrosomes. Interaction of Tax with RanBP1 dysregulates the centrosome duplication pathway resulting in cells with two or more centrosomes following mitosis. **(C) Tax1BP2**. During interphase Tax (green) is expressed in the nucleus and a portion co-localizes with pericentrin (purple) in centrosomes. During S phase centrosomes are duplicated. However, the interaction of Tax with Tax1BP2 (yellow) disrupts the normal controls that prevent centrosome re-duplication resulting in cells with more than two centrosomes. During mitosis, supernumerary centrosomes are separated into two daughter cells resulting in a percentage of cells with two or more centrosomes.

Centrosome amplification, which results in aneuploidy, is seen in a large number of cancers, including adult T cell lymphoma, and the HTLV-1 transactivator Tax plays a critical role in this process. Tax targets several pathways to induce uncontrolled cell growth including Rb [27], cyclin D2 [25,26,41-43], and Cdk 4 [44-48]. However, development of ATL takes decades and only a small percentage of infected individuals' progress to ATL, indicating that additional genetic alterations are required. Interestingly, while lymphoma and acute stage ATL patients display a high amount of structural (clastogenic) and numerical (aneugenic) damage, earlier stage patients (smoldering/chronic) do exhibit some aneugenic damage as exhibited by multinucleated T cells. This suggests that aneugenic damage is an early phenomenon in ATL development; however, how aneugenic damage contributes to malignant transformation is still not known. Duplication of centrosomes and proper segregation of chromosomes into daughter cells is a highly regulated process involving a number of centrosome proteins, which have yet to be fully elucidated. Recent work has demonstrated that Tax can induce genomic instability and aneuploidy by directly targeting several proteins involved in centrosome duplication [Figure 1]. Interaction with Tax1BP2 may interrupt the intrinsic block to centrosome reduplication allowing for centrosome amplification. As mentioned by the authors, future studies will entail determining the mechanism of Tax1BP2 contributing to this block and how Tax may modulate this effect. Since Tax1BP2 appears to be phosphorylated and possibly regulated by Cdk2, Tax may bring Cdk2 into contact with Tax1BP2. Interaction with members of the APC induces a delay in mitotic entry and increased chromosomal instability. The centrosome amplification and chromosomal instabilities would allow for additional genetic alterations. Lastly, the interaction of Tax with Mad1, a component of the mitotic spindle checkpoint, inactivates this critical checkpoint allowing cells with supernumerary chromosomes and improperly segregated chromosomes to move through mitosis and into the next cell cycle. Additional questions remain as to whether there are Tax-dependent defects within cytokinesis that contribute to aneugenic damage and how the three Tax binding proteins identified by this group function together to regulate centrosome duplication. The role of Tax as a transcriptional regulator may also provide avenues for Tax to disrupt mitotic processes. Future studies will not only allow for better understanding of Tax involvement at mitosis but help to further elucidate how deregulation of this phase of the cell cycle contributes to carcinogenesis.

## Competing interests

The author(s) declare that they have no competing interests.

## Authors' contributions

AP and CF did the background research and most of the writing. FK edited and contributed to the figure development.
